# Production of secretory cutinase by recombinant *Saccharomyces cerevisiae* protoplasts

**DOI:** 10.1186/s40064-016-1806-4

**Published:** 2016-02-24

**Authors:** Hideki Aoyagi, Yoichi Katakura, Akio Iwasaki

**Affiliations:** Faculty of Life and Environmental Sciences, University of Tsukuba, Ibaraki, 305-8572 Japan; Clinical Research Support Center, Juntendo University, 2-1-1 Hongo, Bunkyo-ku, Tokyo, 113-8421 Japan

**Keywords:** Cutinase, Immobilization, Protoplast, Recombinant yeast

## Abstract

**Background:**

During heterologous protein production using recombinant microbes, the protein tends to accumulate in the cell and may not be secreted. Here, we studied the production of secretory cutinase (heterologous protein) by recombinant *Saccharomyces cerevisiae* protoplasts.

**Findings:**

Recombinant *S. cerevisiae* cells (i.e., cells into which the cutinase gene was transferred) secreted trace amounts of cutinase into the broth. Approximately 28 % of the cutinase produced in the cells localized to the cell walls and/or between cell wall and cell membrane (CW). In comparison with cell culture, protoplasts in a static culture secreted measurable amounts of cutinase into the broth. Protoplasts were protected from physical and osmotic stresses by entrapping them in a membrane capsule with a low-viscous liquid-core of 1.92 % w/v calcium-alginate. To increase secretory cutinase production, the entrapped protoplasts were cultivated in a shake flask at low osmotic pressure without disruption. During 60 h of cultivation, the extracellular cutinase activity of the free protoplasts at 29.3 atm and protoplasts entrapped in the capsule at 17.2 atm were 0.13 and 0.39 U/mL, respectively.

**Conclusions:**

This is the first report which demonstrates that the efficient production of a secretable enzyme by using protoplasts isolated from recombinant microbes. This system described here is useful to produce products that accumulate in the CW.

**Electronic supplementary material:**

The online version of this article (doi:10.1186/s40064-016-1806-4) contains supplementary material, which is available to authorized users.

## Findings

### Background

Plant and microbial protoplasts are used in genetic transformation and cell fusion techniques. We have studied the use of plant and microbial protoplasts to produce useful metabolites (Tanaka et al. 2000; Mera et al. 2004; Aoyagi 2009 and references therein; Aoyagi et al. 2012). In cases where the cell wall limits the secretion of useful metabolites, production systems using protoplasts can be valuable. One advantage of using protoplasts is that the products are released into the culture broth, resulting in an increase in overall productivity and facilitating down-stream processing. We are currently studying the removal of microbial and plant cell walls to avoid accumulation in the periplasm and to enhance the mass transfer of large quantities of useful metabolites (Aoyagi 2009 and references therein; Aoyagi et al. 2012). It has also been reported that the protoplasts can be used for improving the secretion of native yeast enzymes (Tanaka et al. 2000; Mera et al. 2004).

In the case of recombinant protein production, secretion of the heterologous protein is one of the bottlenecks (Passolunghi et al. 2010 and references therein). Bottlenecks during secretion are often located in the secretory pathway (mainly folding), but may also be related to cell wall retention. In order to overcome the problem, several approaches have been attempted such as improvement of the heterologous gene expression levels, signal sequence optimization, co-expression of chaperones and foldases, preventing intracellular degradation or introduction of mutations which improve secretion capabilities (reviewed by Idiris et al. 2010; Hou et al. 2012; Delic et al. 2014). In the case where the cell wall limits the excretion of the heterologous protein, deletion of cell wall modifying enzymes such as gas1 have been reported (Chow et al. 1992; Vai et al. 2000; Bartkeviciute and Sasnauskas 2004; Passolunghi et al. 2010 and references therein). Alternatively, a production system that uses protoplasts may be effective. However, to our knowledge, there have been no reports on effective recombinant protein production using protoplasts isolated from recombinant microbes, until now.

In this study, we examined the utility of protoplasts for the production of secretory cutinase by using recombinant *Saccharomycescerevisiae* as a model system for cutinase production.

### Methods

#### Preparation of cutinase cDNA

Restriction enzymes, a DNA Blunting Kit, and an *Xho*I linker were purchased from Takara Bio Inc., Shiga, Japan. The cDNA of the cutinase gene (*cutL*) of non-phytopathogenic and industrially important fungus, *Aspergillus oryzae* (Ohnishi et al. 1995), which play an important role in flavor formation during fermentation, was used in this study. The *cutL* was prepared from the p8EHL plasmid (a gift from Prof. Junichi Sekiguchi, Shinshu University) as follows. p8EHL was digested with *Hin*dIII and the formed 3′ recessed end was filled-in with the DNA Blunting Kit. The blunt-ended p8EHL was ligated with the 5′-phosphorylated *Xho*I linker (5′-CCTCGAGG-3′) by using Ligation Solutions A and B in the kit. The *Xho*I linker-ligated plasmid was then digested with *Eco*RI and *Xho*I. The *cutL* cDNA fragment was isolated by 1 % agarose gel electrophoresis and purified with a QIAquick Gel Extraction Kit (QIAGEN, Hilden, Germany).

#### Construction of the cutinase expression vector and transformation of yeast

The purified *cutL* cDNA fragment was inserted into the *Eco*RI and *Xho*I sites of the pYES3/CT vector (Invitrogen, Thermo Fisher Scientific, Waltham, MA, USA, by using ligation solution to construct an expression vector (promoter: *GAL*1, selection marker: *TRP*1, 2 µ origin). *E. coli* DH5α Competent Cells (Takara Bio Inc., Shiga, Japan) were transformed with the ligation products. The nucleotide sequences of the *cutL* cDNA fragment in the constructed expression vector in several transformant clones were confirmed by means of cycle sequencing with the Thermo Sequenase Primer Cycle Sequencing Kit (Amersham, Piscataway, NJ) and an SQ5500E DNA sequencer (Hitachi, Tokyo, Japan). The 5′ upstream sequence of the insert in the expression vector was analyzed using the T7 primer (5′-TAATACGACTCACTATAGGG-3′). To analyze the 3′ downstream sequence of the insert, a PCR-amplified fragment was prepared with the T7 primer and the primer *cutL* up (5′-CGATTTAGGTGACACTATAGATGTAAGCGTGACATAACTA-3′). The nucleotide sequences of the PCR products were analyzed by using the SP6 primer (5′-ATTTAGGTGACACTATAG-3′). The sequence-confirmed expression vector was purified with a QIAprep Spin Miniprep Kit (QIAGEN) and designated as pYES/EXL. *Saccharomyces cerevisiae* INVSc1 was transformed with pYES/EXL according to the small scale-yeast transformation protocol (Invitrogen, Thermo Fisher Scientific). As a cutinase non-producing control, yeast were also transformed with pYES3/CT. The transformants were selected on agar plates of a SC-W minimal medium (Additional file 1: Table S1) supplemented with 20 g/L glucose. After cultivation at 30 °C for 24 h, each colony of the selected transformants was inoculated into 50 mL of SC-W medium supplemented with 20 g/L glucose. The cultivation was carried out in 250-mL flasks shaken at 200 rpm with a rotary shaker at 30 °C for 24 h. Total DNA from the cultured yeast transformants was extracted and purified with DNeasy (QIAGEN). The presence and sequence of the *cutL* insert in the DNA extract were detected by means of PCR and cycle sequencing, respectively.

#### Expression of cutinase in yeast transformants

Cells of *S. cerevisiae* INVSc1 bearing pYES/EXL (or pYES3/CT as a control) were inoculated into 100 mL of SC-W medium supplemented with 20 g/L glucose in 500-mL flasks and shaken at 200 rpm with a rotary shaker at 30 °C for 17 h. An aliquot of the culture was transferred to 100 mL of SC-W supplemented with 20 g/L galactose to generate an initial *A*_600_ of 0.4. Induction of cutinase expression was performed in 500-mL flasks shaken at 200 rpm with a rotary shaker at 30 °C. The growth was monitored by determining the *A*_600_. A 5-mL aliquot of the culture was removed and centrifuged at 1000×*g* for 10 min at 4 °C. The crude extracellular cutinase was obtained as the supernatant. The harvested cells were treated with 6 U/mL Zymolyase 20T (Seikagaku Kogyo, Tokyo, Japan) at 30 °C for 30 h. The enzyme-treated cells were then harvested by centrifugation at 500×*g* for 10 min at 4 °C and washed twice with 50 mM potassium phosphate buffer (pH 7.0) supplemented with 0.9 M mannitol. The washed cells were suspended in 50 mM potassium phosphate buffer (pH 7.0) and sonicated three times at maximum intensity for 15 s by using the Branson Sonifier 150 (Branson Japan, Kanagawa, Japan). The sonicated cell solution was centrifuged at 11,000×*g* for 15 min at 4 °C to obtain crude intracellular cutinase solution.

#### Protoplast isolation

Zymolyaze 20T was used for protoplast preparation from *S. cerevisiae* cells in the logarithmic growth phase. The method for isolating *S. cerevisiae* protoplast was described previously (Aoyagi et al. 2012). Protoplast formation was confirmed by microscopic examination. The protoplast or cell concentration was measured using a hemocytometer (Kayagaki Irikakogyo, Tokyo, Japan). More than 98 % of cells converted into protoplasts. Free protoplasts or cells were cultured statically at 30 °C to allow cutinase production. The initial protoplast or cell concentration in a 300-mL Erlenmeyer flask containing 50 mL of fresh medium was 1 × 10^7^ protoplasts or cells/mL. SC-W medium (Additional file 1: Table S1) supplemented with 20 g/L galactose was used for the cell culture, whereas SC-W medium supplemented with 20 g/L galactose, 0.85 M mannitol, and 1.0 μg/mL aculeacin A (an inhibitor of beta-1,3 glucan synthesis) was used for the protoplast culture to maintain active protoplasts without cell wall regeneration. The aculeacin A (1.0 μg/mL) was added to the broth every 24 h.

#### Immobilization of cells and protoplasts

Cells or protoplasts were immobilized in a 3.0 % w/v agarose gel block by using the method described by Mera et al. (2004). Cells or protoplasts were immobilized in a 2.5 % w/v calcium-alginate gel by using the method of Tanaka et al. (2000). The method of Koyama and Seki (2004a, b) was used to entrap cells or protoplasts in the low-viscous liquid-core of a 1.92 % w/v calcium-alginate membrane capsule prepared by using polyethylene glycol. The viable protoplasts in the membrane capsule were examined by using fluorescence microscopy DMRXA/RD (Leika Mikroscopie und Systeme GmbH, Wetzlar, Germany) after dissolving the gel in sodium hexamethaphosphoric acid and staining with fluorescein diacetate (Wako Pure Chemical Industries, Osaka, Japan).

#### Analytical methods

The residual galactose concentration in the supernatant of the culture fluid was detected with an F-kit for Lactose/D-Galactose (Roche Diagnostics K.K., Tokyo, Japan). Cutinase activity was measured in a reaction mixture containing 0.56 mM *p*-nitrophenylbutyrate, 50.0 mM potassium phosphate buffer (pH 7.0), 11.3 mM sodium cholate, and 0.23 M tetrahydrofuran. The reaction mixture was preheated at 37 °C for 5 min. A 20-μL aliquot of the crude cutinase solution was then added to 980 μL of the preheated reaction mixture and mixed. The *A*_400_ of the reaction mixture was monitored for 1 min. Units of enzyme activity were determined from the *A*_400_ by using a calibration curve. One unit (U) of cutinase activity was defined as the amount of the enzyme that produced 1 μmol of *p*-nitrophenol from *p*-nitrophenylbutylate in 1 min. All experiments were performed in triplicate. The results are expressed as means; there was less than 5 % deviation in the results.

### Results and discussion

#### Characteristics of *S. cerevisiae* cell and protoplast transformants

**Fig. 1:**
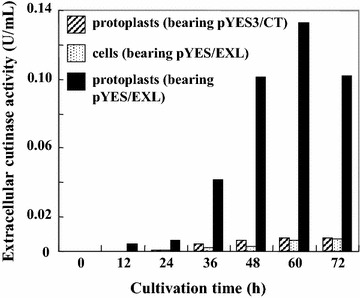
Time course of the extracellular cutinase activity of cultured *S. cerevisiae* cells bearing pYES/EXL, protoplasts bearing pYES/EXL, and protoplasts bearing pYES3/CT. All experiments were performed in triplicate. The results are expressed as means; there was less than 5 % deviation in the results. In the cell culture at 72 h, the *A*_600_ was 6.0

In the *S. cerevisiae* transformant cells (bearing pYES/EXL), there was minimal detectable extracellular cutinase activity during cultivation, whereas intracellular cutinase activity was detected after 24 h of cultivation and reached a maximum value (0.10 U/mL) at 48 h of cultivation. To investigate the localization of the cutinase in these yeast transformant cells, we made use of a protoplast isolation reaction. The cutinase activity detected during the protoplast isolation reaction was considered to be the same as that located within the cell wall and/or between the cell wall and cell membrane (CW). The cutinase activity detected by the disruption of protoplasts was considered to be the same as that in the cytoplasm. In the yeast transformant cells at 48 h of cultivation, approximately 28 % of the cutinase (on the basis of cutinase activity) in the cells was located in the CW. Since some cutinase was located in the CW and cutinase activity was not detected in the medium, it is possible that the CW limited the secretion of the cutinase. Therefore, given that removal of the cell walls might enhance cutinase production, we developed a production system involving isolated protoplasts. Figure 1 shows the extracellular cutinase production by the yeast transformant cells and protoplasts in a static culture. For the *S. cerevisiae* cells (bearing pYES/EXL) and protoplasts (bearing pYES3/CT), extracellular cutinase activity was minimal during cultivation (the intracellular cutinase activity of the cells was about 0.07 U/mL). In the case of the *S. cerevisiae* protoplasts (bearing pYES/EXL), extracellular cutinase activity was detected after 36 h of cultivation and reached a maximum value (0.13 U/mL) at 60 h of cultivation (the intracellular cutinase activity of the protoplasts was about 0.04 U/mL). In the protoplasts culture, about 76 % of the cutinase (on the basis of cutinase activity) was found in the culture filtrate. In the case of cells, cell concentration (cells/mL) increased about 14.5 times after 60 h cultivation, while protoplast concentration did not change. Specific cutinase activity was calculated as the cutinase activity divided by the cell or protoplast concentrations. The specific cutinase activity of the protoplasts (1.70 × 10^−8^ U/protoplast) at 60 h of cultivation was 35.4-fold higher than that in the cell culture (0.46 × 10^−9^ U/cell). Thus, it was clear that cutinase could be efficiently produced extracellularly by the protoplasts. Mera et al. (2004) reported that certain genes associated with the secretory pathway of metabolites are activated in cultured *S. cerevisiae* protoplasts, leading to the active secretion of some metabolites into the broth. A similar mechanism may be associated with the secretory cutinase production described in the present study.

#### Secretory production of cutinase by immobilized recombinant yeast protoplasts

**Fig. 2:**
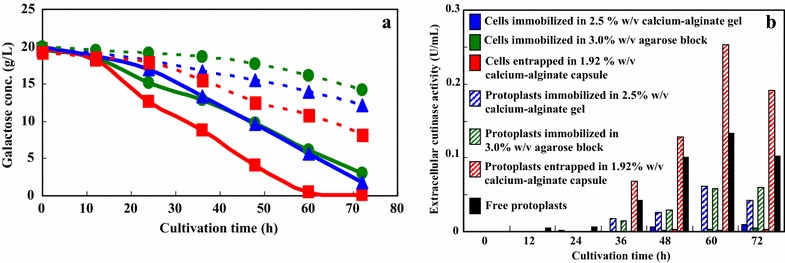
Time course of the galactose consumption (**a**) and extracellular cutinase activity (**b**) of *S. cerevisiae* cells and protoplasts (bearing pYES/EXL) immobilized in various kinds of gel in suspension culture. All experiments were performed in triplicate. The results are expressed as means; there was less than 5 % deviation in the results. *Symbols*: *blue triangles* with a *solid line*, cells immobilized in a 2.5 % w/v calcium-alginate gel; *green circles* with a *solid line*, cells immobilized in a 3.0 % w/v agarose block; *red squares* with a *solid line*, cells entrapped in a 1.92 % w/v calcium-alginate capsule; *blue triangles* with a *dotted line*, protoplasts immobilized in a 2.5 % w/v calcium-alginate gel; *green circles* with a *dotted line*, protoplasts immobilized in a 3.0 % w/v agarose block; *red squares* with a *dotted line*, protoplasts entrapped in a 1.92 % w/v calcium-alginate capsule

**Fig. 3:**
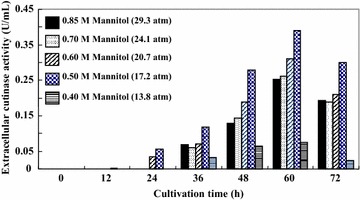
Effect of the osmotic pressure of the medium on the extracellular production of cutinase by *S. cerevisiae* protoplasts (bearing pYES/EXL) entrapped in a 1.92 % w/v calcium-alginate capsule. All experiments were performed in triplicate. The results are expressed as means; there was less than 5 % deviation in the results

Protoplasts are very fragile and sensitive to physical shock and osmotic stress; they are also very difficult to maintain in shake cultures. The stability of protoplasts can be improved by immobilizing them in various kinds of immobilization carriers. They can then be cultivated in shaking at 150 rpm without disruption, as confirmed by microscopic observation and fluorometry. Figure 2 shows the time courses of the galactose consumption and extracellular cutinase activity of cells and protoplasts immobilized in various kinds of immobilization carriers. The galactose consumption by the cells and protoplasts entrapped in the low-viscous liquid-core of a calcium-alginate (1.92 % w/v) membrane capsule was greater than that by cells and protoplasts in other immobilization carriers. In the case of the immobilized cells, the extracellular cutinase activity was minimal during cultivation. In the case of the immobilized protoplasts, the extracellular cutinase activity was detected after 36 h of cultivation; however, the extracellular cutinase activity of the protoplasts immobilized in the 2.5 % w/v calcium-alginate gel and in the 3 % w/v agarose block was lower than that of free protoplasts. In contrast, the extracellular cutinase activity of the protoplasts entrapped in the low-viscous liquid-core of an alginate membrane capsule was 0.25 U/mL at 60 h (i.e., about 2 times that of free protoplast). This capsule has a hollow structure, and the membrane is thin, hence there is the opportunity for better mass transfer relative to the other immobilization carriers. Koyama and Seki (2004b) reported that the apparent effective diffusivity of glucose into the capsule was 7.9 × 10^−10^ m^2^/s, which is larger than that into alginate beads (6.5 × 10^−10^ m^2^/s) or into water (6.7 × 10^−10^ m^2^/s). On the basis of these results, it is apparent that the capsule is a superior immobilization carrier and hence could be useful for the production of secretory cutinase. To prevent the disruption of free protoplasts, the osmotic pressure of the medium must be maintained higher than for free cells. High osmotic pressure of medium has been reported to inhibit the production of enzymes and secondary metabolites by plant cells and protoplasts (Zhao et al. 2000; Mera et al. 2003). However, protoplasts are stable only under high osmotic pressure. Since immobilization can protect protoplasts from disruption under lower osmotic pressure, we investigated cultivation of immobilized protoplasts under low osmotic pressure conditions with the aim of increasing cutinase productivity. Figure 3 shows the effect of the osmotic pressure of the medium on the extracellular production of cutinase by *S. cerevisiae* protoplasts (bearing pYES/EXL) entrapped in the 1.92 % w/v calcium-alginate capsule. The protoplasts entrapped in the capsule could be cultivated under a wide range of osmotic pressures (from 17.2 to 29.3 atm). The extracellular cutinase activity of the protoplasts entrapped in the capsule increased with decreasing osmotic pressure and reached a maximum value (0.39 U/mL) at 17.2 atm and 60 h of cultivation. Most protoplasts were viable, which was confirmed by fluorescence microscopic observation. This was three times that of free protoplasts at 29.3 atm. The increased cutinase production under low osmotic pressure could be due to water movement into the protoplasts under low osmotic pressure conditions, increased physical stress on the protoplasts, and/or cutinase secretion from the protoplasts. A similar phenomenon has been reported in plant protoplasts (Mera et al. 2003). We are currently investigating the mechanism of this phenomenon. The cutinase productivity described here is still very low for practical and commercial application. In order to enhance the productivity, the optimization of culture condition and construction of continuous production system of cutinase by protoplasts entrapped in the capsule coupled with simultaneous product recovery from the medium, etc. are necessary. Several researchers have reported the production of cutinase from phytopathogenic fungus with higher productivities (Calado et al. 2002, 2003; Kwon et al. 2009; Seman et al. 2014; Nyyssola 2015). It has also been reported that the cutinase have great potential for applications in the textile industry, laundry detergents, the processing of biomass and the biocatalysis and detoxification of environmental pollutants (Nyyssola 2015). These reported approaches may be useful to enhance the productivity of cutinase from non-phytopathogenic fungus described in this paper.

In conclusion, this is the first report that demonstrates that the efficient production of a secretable enzyme by using protoplasts isolated from recombinant microbes. During heterologous protein production using recombinant microbes, some proteins tend to accumulate in the cell and may not be secreted. In such cases, a production system that uses protoplasts is effective. We believe that this system may be useful to produce other products that accumulate in the CW.

## Additional file

10.1186/s40064-016-1806-4 Composition of SC-W minimal medium (pH 6.0).

## Abbreviation

CW: cell wall and/or between the cell wall and cell membrane
