# Supplementary material for: Production of secretory cutinase by recombinant Saccharomyces cerevisiae protoplasts
Source: Springerplus. 2016 Feb 24;5:160. doi: 10.1186/s40064-016-1806-4 (PMC4766152; doi:10.1186/s40064-016-1806-4)
Supplement: Supplementary file 1 — 10.1186/s40064-016-1806-4 Composition of SC-W minimal medium (pH 6.0). [file 40064_2016_1806_MOESM1_ESM.docx]

Table S1 Composition of SC-W minimal medium (pH 6.0)

| Component | (g/L) |
| --- | --- |
| YNB without amino acids (with ammonium sulfate)* | 6.70 |
| Adenine | 0.10 |
| Arginine | 0.10 |
| Cysteine | 0.10 |
| Leucine | 0.10 |
| Lysine | 0.10 |
| Threonine | 0.10 |
| Uracil | 0.10 |
| Aspartic acid | 0.05 |
| Histidine | 0.05 |
| Proline | 0.05 |
| Isoleucine | 0.05 |
| Methionine | 0.05 |
| Serine | 0.05 |
| Tyrosine | 0.05 |
| Valine | 0.05 |

*Yeast Nitrogen Base without amino acids (Becton, Dickinson and Company, Franklin Lakes, NJ)
